# Targeting Inflammatory Mediators in Epilepsy: A Systematic Review of Its Molecular Basis and Clinical Applications

**DOI:** 10.3389/fneur.2022.741244

**Published:** 2022-03-11

**Authors:** Giorgio Costagliola, Greta Depietri, Alexandre Michev, Antonella Riva, Thomas Foiadelli, Salvatore Savasta, Alice Bonuccelli, Diego Peroni, Rita Consolini, Gian Luigi Marseglia, Alessandro Orsini, Pasquale Striano

**Affiliations:** ^1^Pediatric Immunology, Pediatric University Department, Azienda Ospedaliero Universitaria Pisana, University of Pisa, Pisa, Italy; ^2^Pediatric Neurology, Pediatric University Department, Azienda Ospedaliero Universitaria Pisana, University of Pisa, Pisa, Italy; ^3^Pediatric Clinic, Istituto di Ricovero e Cura a Carattere Scientifico (IRCCS) Policlinico San Matteo Foundation, University of Pavia, Pavia, Italy; ^4^Pediatric Neurology and Muscular Diseases Unit, IRCCS Istituto “Giannina Gaslini”, Genova, Italy; ^5^Department of Neurosciences, Rehabilitation, Ophthalmology, Genetics, Maternal and Child Health, University of Genova, Genova, Italy

**Keywords:** adalimumab, anakinra, canakinumab, cytokines, neuroinflammation, epilepsy, tocilizumab, rituximab

## Abstract

**Introduction:**

Recent studies prompted the identification of neuroinflammation as a potential target for the treatment of epilepsy, particularly drug-resistant epilepsy, and refractory status epilepticus. This work provides a systematic review of the clinical experience with anti-cytokine agents and agents targeting lymphocytes and aims to evaluate their efficacy and safety for the treatment of refractory epilepsy. Moreover, the review analyzes the main therapeutic perspectives in this field.

**Methods:**

A systematic review of the literature was conducted on MEDLINE database. Search terminology was constructed using the name of the specific drug (anakinra, canakinumab, tocilizumab, adalimumab, rituximab, and natalizumab) and the terms “status epilepticus,” “epilepsy,” and “seizure.” The review included clinical trials, prospective studies, case series, and reports published in English between January 2016 and August 2021. The number of patients and their age, study design, specific drugs used, dosage, route, and timing of administration, and patients outcomes were extracted. The data were synthesized through quantitative and qualitative analysis.

**Results:**

Our search identified 12 articles on anakinra and canakinumab, for a total of 37 patients with epilepsy (86% febrile infection-related epilepsy syndrome), with reduced seizure frequency or seizure arrest in more than 50% of the patients. The search identified nine articles on the use of tocilizumab (16 patients, 75% refractory status epilepticus), with a high response rate. Only one reference on the use of adalimumab in 11 patients with Rasmussen encephalitis showed complete response in 45% of the cases. Eight articles on rituximab employment sowed a reduced seizure burden in 16/26 patients. Finally, one trial concerning natalizumab evidenced a response in 10/32 participants.

**Conclusion:**

The experience with anti-cytokine agents and drugs targeting lymphocytes in epilepsy derives mostly from case reports or series. The use of anti-IL-1, anti-IL-6, and anti-CD20 agents in patients with drug-resistant epilepsy and refractory status epilepticus has shown promising results and a good safety profile. The experience with TNF inhibitors is limited to Rasmussen encephalitis. The use of anti-α4-integrin agents did not show significant effects in refractory focal seizures. Concerning research perspectives, there is increasing interest in the potential use of anti-chemokine and anti-HMGB-1 agents.

## Introduction

**Figure 1:**
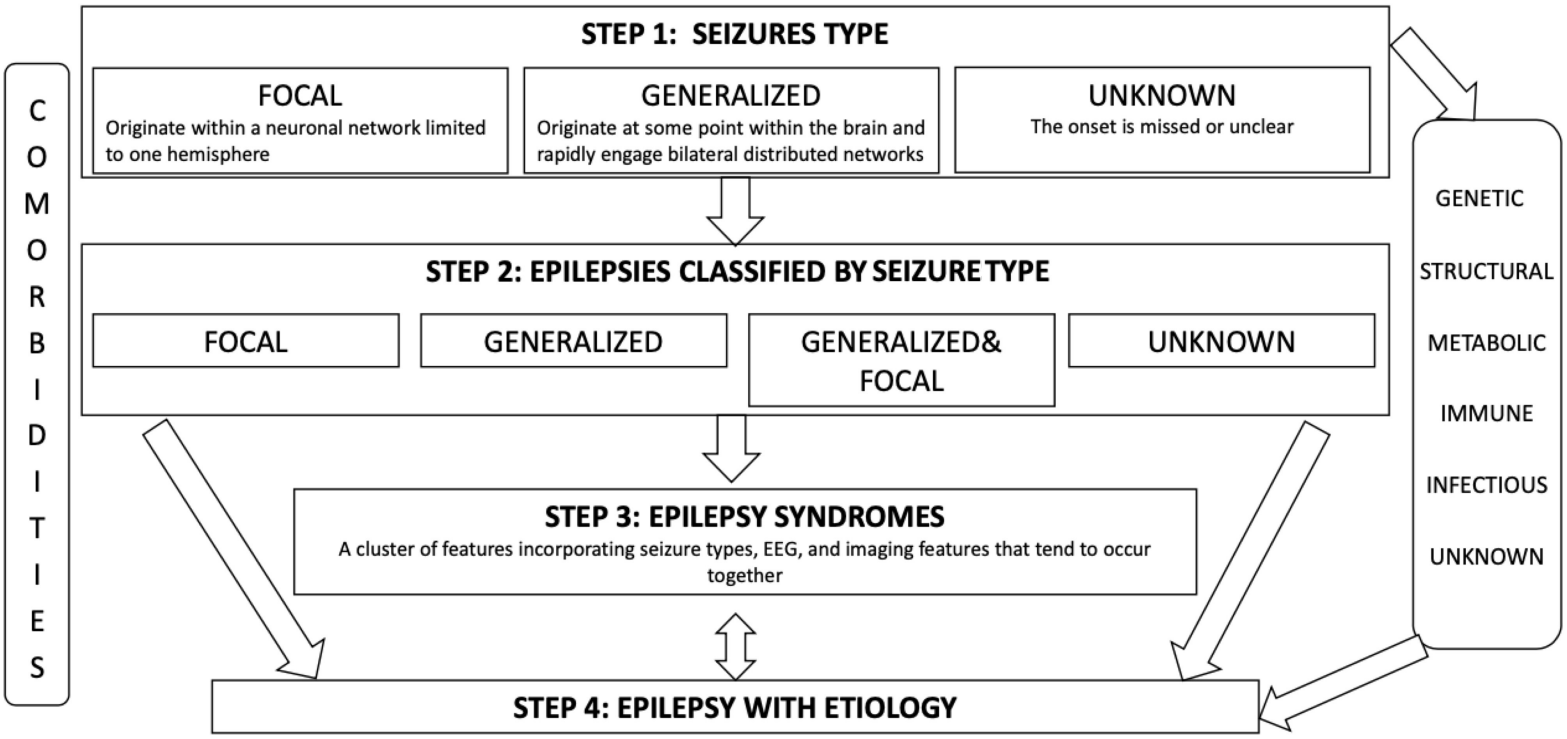
Framework for epilepsy classification designed to allow diagnosis at multiple levels depending on the informationand resources available. At all levels of diagnosis, we should consider more broadly the etiology of the patient's epilepsy. A range of six etiological groups has been recognized: genetic, structural, metabolic, immune, infectious, and unknown.

Epilepsy affects more than 50 million people worldwide and represents an unsolved public health problem (1). In 2017 ILEA published an operational classification of seizures ad epilepsies which gave a remarkable contribution in improving epilepsy diagnosis and treatment (2, 3) (Figure 1). Despite the significant advances made in its treatment, about 7–20% of the children and 30–40% of the adults develop drug-resistant epilepsy (DRE) (4), which can be reasonably defined as an incompletely controlled disease despite the trial with two appropriate antiseizure medications (ASM; whether as monotherapy or in combination) at the correct posology) (5, 6). One of the most severe clinical complications observed in epileptic individuals is status epilepticus (SE), defined by ILEA (7) (Table 1). Refractory status epilepticus (RSE) is defined by a SE in which the administration of a benzodiazepine bolus and another ASM does not resolve the clinical picture (13). Among the research areas to improve the therapeutic strategy against drug-resistant epilepsy and RSE, there is increasing interest in the role of agents targeting neuroinflammation. Neuroinflammation is a non-specific biologic response of the brain and spinal cord innate immune system (14), which shows a bidirectional relationship with seizures. Indeed, seizures themselves can be responsible for neuronal and glial damage followed by an inflammatory response, while experimental studies investigating the effects of the administration of pro-inflammatory molecules in the central nervous system (CNS) evidenced that inflammation significantly reduces the seizure threshold (15). Although different authors have focused on the identification of the molecular mechanisms responsible for the inflammatory-dependent induction of seizures and the research for potential therapeutic agents targeting neuroinflammation for the treatment of epilepsy (16, 17), there is no uniform agreement on their use, and data from large cohorts of patients and randomized studies are missing.

**Table 1:** Definitions.

**Operational (practical) clinical definition of epilepsy (8)**
Epilepsy is a disease of the brain defined by any of the following conditions: • 1. At least two unprovoked (or reflex) seizures occurring >24 h apart • 2. One unprovoked (or reflex) seizure and a probability of further seizures similar to the general recurrence risk (at least 60%) after two unprovoked seizures, occurring over the next 10 years • 3. Diagnosis of an epilepsy syndrome
Epilepsy is considered to be resolved for individuals who had an age-dependent epilepsy syndrome but are now past the applicable age or those who have remained seizure-free for the last 10 years, with no seizure medicines for the last 5 years.
**Status epilepticus (7)**
“A condition resulting either from the failure of the mechanisms responsible for seizure termination or from the initiation of mechanisms, which lead to abnormally, prolonged seizures (after time point t_1_). It is a condition, which can have long-term consequences (after time point t_2_), including neuronal death, neuronal injury, and alteration of neuronal networks, depending on the type and duration of seizures.”
**Super refractory status epilepticus (7)**
SE continues for more than 24 h after the first administration of general anesthesia.
**Autoimmune encephalitis (9)**
Autoimmune encephalitis encompasses a wide variety of protean pathologic processes associated with the presence of antibodies against neuronal intracellular proteins, synaptic receptors, ion channels and/or neuronal surface proteins.
**Rasmussen encephalitis (10)**
Unilateral hemispheric encephalitis whose main clinical features include refractory focal epilepsy or epilepsia partialis continua, hemiparesis, and progressive cognitive decline.
**New-onset refractory status epilepticus (NORSE) (11)**
NORSE is a clinical presentation, not a specific diagnosis, in a patient without active epilepsy or other preexisting relevant neurological disorder, with new onset of refractory status epilepticus without a clear acute or active structural, toxic or metabolic cause.
**Febrile infection-related epilepsy syndrome (FIRES) (11)**
FIRES is a subcategory of NORSE, applicable for all ages, that requires a prior febrile infection starting between 2 weeks and 24 h prior to onset of refractory status epilepticus, with or without fever at onset of status epilepticus.
**Electrical status epilepticus in sleep (ESES) (12)**
Electrical status epilepticus in sleep (ESES), a childhood-onset epileptic encephalopathy, is characterized by epilepsy, cognitive regression, and marked activation of epileptiform activity during non-rapid eye movement (NREM) sleep to produce an electroencephalography (EEG) pattern of near-continuous spike-wave discharges.

In this work, we review the known molecular mechanisms linking neuroinflammation and epilepsy, including the role of the main inflammatory mediators involved in the process of epileptogenesis. For epileptic syndromes cited and terminology related to epilepsy we used the new ILEA operational practical clinical definition of epilepsy (Table 1) (8). Moreover, we provide a systematic review of the clinical experience with anti-cytokine agents (anti-IL-1, anti-IL-6, and anti-TNF agents) and with agents targeting the effectors of adaptive immunity in the treatment of epilepsy, with a focus on DRE and RSE. Finally, in the last section of this work, we discuss the main research perspectives in this field, including anti-chemokine agents. This paper aims to help reduce the knowledge gap in the field of neuroinflammation in epileptic individuals and represents the first systematic review performed on the role of anti-cytokine and anti-lymphocyte agents.

## Seizures and Neuroinflammation—An Overview

**Figure 2:**
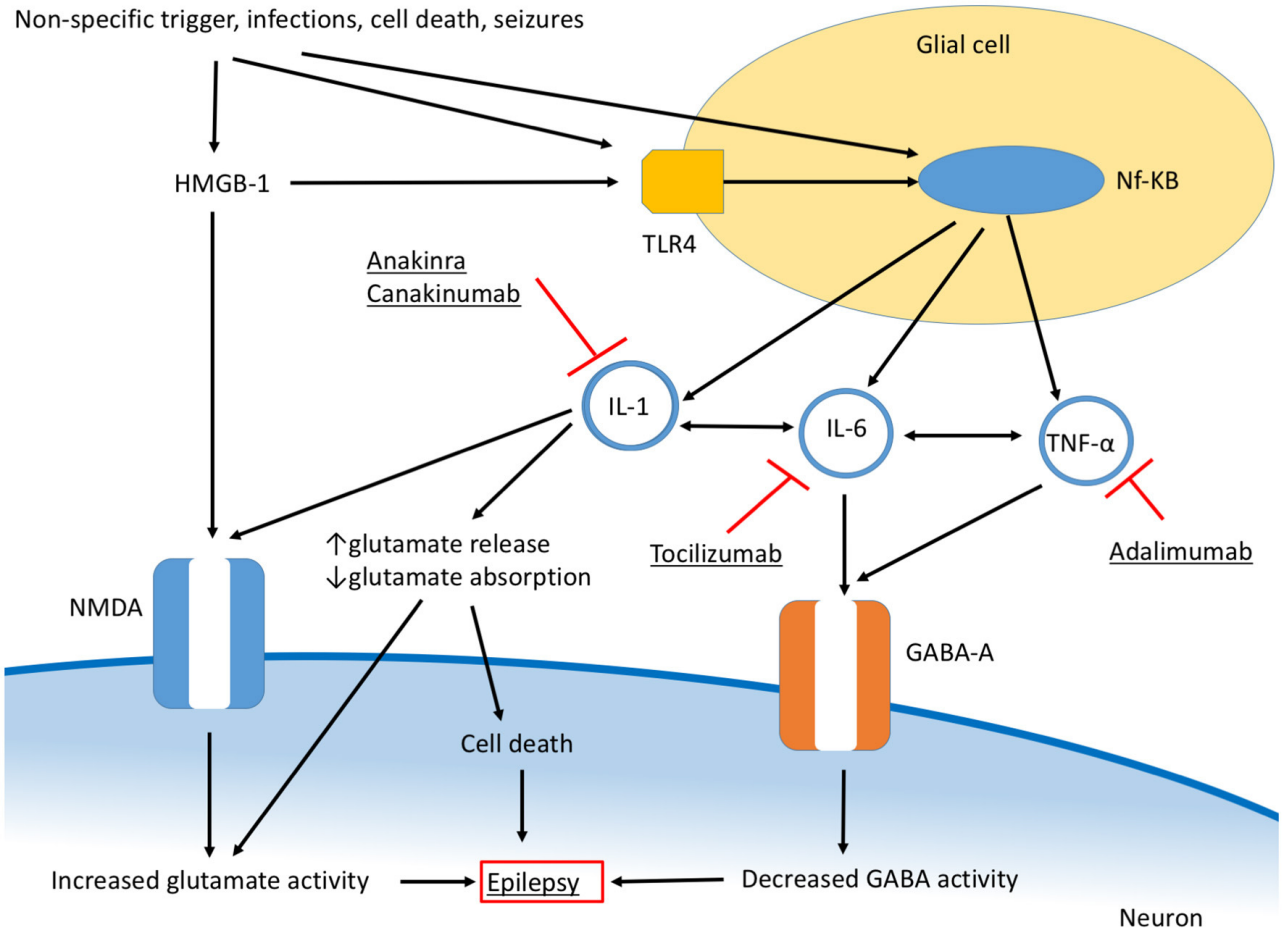
Overview of inflammatory pathways involved in epilepsy and main therapeutic targets.

Neuroinflammation develops as a consequence of different stimuli, such as CNS trauma, infection, ischemic and hemorrhagic diseases, and seizures, although it can be also the consequence of a systemic inflammatory response spreading to CNS (14). Additionally, neuroinflammation is partly responsible for the neuronal damage and clinical manifestations of CNS disorders primarily featured by uncontrolled adaptive immune function, including autoimmune encephalitis (Anti-NMDR, Anti-AMPAR, Anti-GABA/AR and Anti-GABA/BR, Anti-VGKC, Anti-GAD, Anti-GlyR, Anti-DPPX, Anti-mGluR5, Anti-IgLON5) (18, 19) and Rasmussen syndrome (10) (Table 1), and others. Although inflammatory mediators are central in maintaining brain homeostasis, being implicated in the initiation of tissue repair after CNS injury, the process of neurogenesis, neuronal plasticity, and in the behavioral responses related to stress, neuroinflammation can be responsible for neuronal damage, altered cellular function, and impaired secretion and response to neurotransmitters, thus participating to the process of epileptogenesis (14). The CNS inflammatory response depends on the activity of the resident innate immune cells (particularly, microglial cells and astrocytes) and the release of inflammatory mediators, including cytokines, chemokines, prostaglandin, nitric oxide (NO), and reactive oxygen species (ROS) (17). Among the cellular effectors of neuroinflammation, microglial cells play a central role. They function as sensors for different triggers, having macrophage-like activity, and are implicated in the recognition of pathogens and cellular debris, and the secretion of cytokines and chemokines (20). The function of immune surveillance of microglial cells depends on multiple cellular receptors, with toll-like receptors (TLR) being directly involved in the recognition of pathogen-associated molecular patterns (PAMPS) (21). Other cellular subtypes are involved in CNS inflammation and include endothelial cells and perivascular macrophages, which can act increasing vascular permeability and, through the expression of adhesion molecules, contribute to leukocyte chemotaxis (14, 22). The main cytokines participating in the CNS inflammatory response and epileptogenesis are interleukin 1β (IL-1β), IL-6, and tumor necrosis factor-α (TNF-α) (Figure 2). An elevation of the serum and cerebrospinal (CSF) levels of these cytokines in individuals with epilepsy has been demonstrated in different studies (23), as well as the higher expression of genes encoding for these mediators in the brain tissue from patients who underwent brain resection for drug-resistant focal epilepsy (17). Interestingly, the levels of inflammatory mediators are higher in patients with drug-resistant epilepsy (24), suggesting a contribution of neuroinflammation in the development of drug resistance. Additionally, the demonstration that in animal models the intrathecal administration of IL-1β causes a reduction of the seizure threshold pointed out the direct role of cytokines in seizure and epileptogenesis (15).

Among soluble inflammatory mediators, the danger signal molecule high-mobility group box-1 (HMGB-1) also plays a central role in neuroinflammation, being involved in the TLR4-IL-1β axis, and in the enhancement of the CNS inflammatory response (25). Chemokines and their receptors participate in CNS inflammation favoring leukocyte migration to the site of inflammation and endothelial adhesion but are also directly implicated in epileptogenesis through the interaction with neurotransmitters and neuropeptides (26), as further discussed. Finally, the role of the blood-brain barrier (BBB) in neuroinflammation and epileptogenesis is of extreme clinical and research interest (25). Inflammatory mediators, produced both in CNS and systemically, can enhance its permeability and BBB leakage allows the spread of systemic inflammatory mediators within the CNS, thus amplifying the inflammatory process (17).

### IL-1 and Epilepsy: Molecular Basis

IL-1β is mostly produced by cells of the innate immune system, including monocytes, macrophages, and dendritic cells, following a wide range of triggers, such as infections and cellular damage (i.e., oxidative stress) (27). In CNS, IL-1β is produced by glial cells and other cellular sources, including endothelial cells (28). The main molecular mechanism leading to the synthesis of IL-1β is the assembly and function of the macromolecular complex of the inflammasome, in a process that requires the recognition of the inflammatory trigger (i.e., *via* toll-like receptors) leading to the activation of caspase-1, finally allowing the release of active IL-1β (29). Once released, IL-1β acts as an initiator of the inflammatory response, promoting the synthesis of other pro-inflammatory cytokines (such as IL-6), induces fever, and acts as a direct effector of inflammatory organ damage (30). High serum and CSF levels of IL-1β have been demonstrated in individuals suffering from epilepsy, including those affected by developmental epileptic encephalopathies, with even higher concentrations in patients with drug-resistant epilepsy (17). Although the mechanisms linking IL-1β and epileptogenesis are far to be fully understood, pieces of evidence suggest that neuronal excitation and excitotoxicity secondary to the enhanced effect of glutamate play a significant role (31). Indeed, IL-1β has been demonstrated to influence the calcium influx across the N-methyl-D-aspartate (NMDA) receptor, reduce glutamate uptake by astrocytes and increase glutamate release by glial cells (32). On the other hand, studies on the influence of IL-1β on GABA-ergic transmission show conflicting results (32).

Currently, the agents targeting IL-1β comprise the IL-1 receptor antagonist anakinra, the anti-IL-1 monoclonal antibodies canakinumab (human antibody) and gevokizumab (humanized antibody), and the IL-1 inhibitor rilonacept, which consists of a fusion protein composed of the Fc portion of human IgG and the extracellular domain of IL-1 receptor (33, 34). Since their introduction, anti-IL-1 agents have become a cornerstone in the treatment of autoinflammatory disorders, and have also been used in other rheumatologic disorders, such as rheumatoid arthritis (RA), gout, adult-onset Still diseases and systemic juvenile idiopathic arthritis (sJIA), and cytokine storm syndromes (35), with anakinra and canakinumab being the most widely used drugs. The use of gevokizumab is still experimental, since the drug has not been approved by the Food and Drug Administration.

### IL-6 and Epilepsy: Molecular Basis

IL-6 has a pivotal role in enhancing and maintaining the inflammatory response and activating adaptive immunity. Systemically, the release of IL-6 is followed by the production of acute-phase proteins (C-reactive protein, serum amyloid protein, fibrinogen), the release of platelets, angiogenesis, and an increase in vascular permeability (36). Additionally, IL-6 participates in driving the differentiation of T CD4+ cells in T helper 17 (Th17) cells, promotes the differentiation and expansion of T CD8+ cytotoxic cells, and inhibits the proliferation of regulatory T cells (Tregs) (36). In the CNS, this cytokine is mostly produced by glial cells, with a mechanism triggered by multiple factors, including the binding of IL-1β and TNF-α to specific surface receptors on glial cells (37). Additionally, IL-6 can be secreted by astrocytes and neurons and can be released by perivascular and brain endothelial cells in response to inflammatory and infectious stimuli (37). Increased serum and CFS levels of IL-6 have been demonstrated in patients with refractory epilepsy, and an association between IL-6 levels and the degree of neuronal apoptosis was evidenced in patients undergoing surgery for refractory temporal lobe epilepsy (38). Notably, the serum cytokine levels have been shown to decrease after surgery (38). Apart from the induction of apoptosis, a direct role of IL-6 in epileptogenesis has been suggested (31). It is known that IL-6 interferes with GABA-A receptor functioning, while recent data suggest that the cytokine could reduce glutamate signaling and consequent neuronal toxicity (32).

The most widely used anti-IL-6 therapeutic agent is tocilizumab, a humanized monoclonal anti-IL-6 receptor antibody that is part of the therapeutic armamentarium against autoinflammatory disorders, cytokine storm syndromes, and autoimmune diseases (36, 39). Concerning neurological diseases, tocilizumab is used in autoimmune encephalitis and different systemic inflammatory disorders with CNS involvement, including linear scleroderma “*en coup de sabre*” (LSCS), and neuro-Behcet's disease (40, 41). The human monoclonal anti-IL-6 antibody sarilumab is a therapeutic option in patients with RA and different authors suggested a role in the treatment of severe COVID-19 (42, 43).

### TNF-α and Epilepsy: Molecular Basis

Tumor necrosis factor-alpha (TNF-α) is a pleiotropic effector cytokine of the TNF superfamily that plays a role in the regulation of cell homeostasis and of the immune-inflammatory pathways (28). TNF-α is produced by a wide range of cells (including microglia, astrocytes, and neurons) (44, 45) and interacts with two transmembrane glycoprotein receptors, TNF receptor 1 (TNFR1) and TNF receptor 2 (TNFR2), that differ in their cellular expression profiles, ligand affinities and signaling pathways (45). The main downstream effectors are Nuclear Factor Kappa-B, C-Jun N-terminal Kinase, p38, and the pathway of sphingomyelinase/ceramide. The exact role of TNF-α in epileptogenesis is not yet understood (46). The cytokine may operate by direct interaction with neurons or by influencing the expression of neurotransmitter receptors on glial cells (47, 48). TNF-α can also alter the permeability of the blood-brain barrier (28, 49). Reportedly, the expression of TNF-α in astrocytes may promote an inflammatory and degenerative outcome, while the cytokine expression in neuronal and microglial cells would be associated with tissue repair and remyelination (50–52). This dual role of TNF-α reflects the subtype of receptor that is preferentially involved in the signaling: TNFR1 is thought to induce an epileptogenic and proinflammatory phenotype through a series of post-translational mechanisms that regulate the expression and the turnover of AMPA, GABAA, and NMDA-NR1 receptors, while TNFR2 shows an anticonvulsant and neurotrophic orientation. Indeed, a study conducted on hippocampal tissues from patients with intractable temporal lobe epilepsy documented the predominance of TNFR1 pathways. Specifically, the involvement of TNFR1 caused the activation of apoptosis pathways capable to perpetrate the seizure-induced brain injury (53).

All molecules with TNF-inhibitory action that are currently approved for therapeutic use are monoclonal antibodies (mAbs) obtained by mutation and gene-splicing techniques (54, 55). These drugs can act both with an antagonistic effect, blocking the cellular functions mediated by TNF-receptors, or with an agonistic function through reverse signaling mediated by the transmembrane portion of TNF-α (56). In the spectrum of CNS disorders, the use of anti TNF-α agents has been specifically studied for multiple sclerosis (MS) (57, 58). However, two clinical trials evidenced discouraging results, since patients showed clinical and radiological signs of progression of the disease during treatment (57, 58). Moreover, a possible correlation between the exposure to anti-TNF-α agents and the occurrence of inflammatory demyelinating and non-demyelinating events emerges from different cases and case-series of patients treated for non-CNS related disorders (59–69). We speculate that the adverse neurological effects observed in patients treated with TNF-inhibitors for other diseases influence and limit the choice of these therapies for the treatment of many neurological conditions. Despite a causal correlation between demyelination and the use of anti TNF-α agents is uncertain, some mechanisms have been proposed to explain the pathogenesis of the adverse events observed, including the fact that the use of TNF-inhibitors may reduce the expression of TNFR2 receptors within the brain tissues, impairing the course of reparative processes (70–73). In the particular case of epilepsy, animal models highlighted how the effects of the TNF-α can be different depending on which receptor subtype governs the signaling pathway. Hence, future therapeutic agents may be targeted to the mechanisms that govern the predominance between TNFR1 and TNFR2, to downregulate the former without reducing the expression of the latter (74).

### Adaptive Immunity and Epilepsy: Molecular Basis

Adaptive immunity plays an important role in the immune surveillance within the CNS through a series of mechanisms, considering the status of immune privilege of the CNS (75). Nonetheless, cells of adaptive immunity have also been implied in the pathogenesis of different immune-mediated diseases of the CNS (76). Moreover, experimental studies conducted in models of epilepsy suggested that adaptive immunity is actively involved in this process (77). Following seizures, the alteration of the BBB leads to the release of chemokines and drives the infiltration of lymphocytes and other cells of the innate immunity (i.e., monocytes) (78). Within the CNS, B and T cells exert their effector functions with different mechanisms. The understanding of the pathogenesis of certain forms of immune-mediated epilepsy revealed that B and T cells can respond to stimuli originating directly by neuronal antigens or by molecular mimicry between the antigens of infectious agents (mainly viruses) and components of the CNS tissues (79, 80). To date, a wide range of antibodies directed toward extracellular domains have been described and reunited under the term “Neuronal Surface Antibodies.” The presence of such antibodies underlies several CNS syndromes characterized by seizures (81). In other conditions, identified as paraneoplastic syndromes, the production of antibodies is rather an epiphenomenon and the pathogenesis recognizes a role of cytotoxic T cells (82). Xu et al. investigated the involvement of cell-mediated immunity in patients with intractable forms of epilepsy and demonstrated localization of blood-derived, antigen-specific CD8+ and γδ T cells in the epileptogenic zone, showing how the amount of infiltrated cells correlated with seizure severity (83). Interestingly, the majority of T cells detected resulted to be memory T cells, capable to sustain an inflammatory response even in the absence of costimulatory signals. Additionally, the amount of brain infiltrating T reg cells resulted inversely correlated with seizure severity (83). The role of CD8+ cells is peculiar since under inflammatory conditions neurons can express MHC class I and II molecules perpetrating cell-mediated brain damage (84).

Studies conducted in the past years raised the awareness of how targeting the mechanisms of action of B and T cells could be of therapeutic interest. The use of monoclonal antibodies as immune modulators has substantially changed the approach to many inflammatory diseases, including some autoimmune disorders of the CNS (85). The activity of these agents consists mainly in inhibiting the production of antibodies and blocking the intrusion of effector lymphocytes in the CNS. Rituximab is a chimeric anti-CD20 mAb engineered to reduce the pool of B cells that undergo maturation and that produce antibodies, hence decreasing the entity of humoral immune response (86). Originally approved for the treatment of B cell lymphoma, to date rituximab is employed in the treatment of many immune-mediated diseases of the CNS, including multiple sclerosis and other forms of autoimmune demyelinating disorders (87, 88). This molecule represents also an off-label second line of treatment for several forms of autoimmune encephalitis (89). Particularly, the use of rituximab for the treatment of anti-NMDR has been the object of studies that evidenced a good response in terms of outcome and a lower frequency of relapses (19, 90). With a more heterogeneous degree of clinical response, the use of rituximab is also reported in cases of encephalitis associated with anti-GAD, anti-myelin oligodendrocyte glycoprotein, and anti-leucine-rich glioma inactivated 1 (LGI1) antibodies (91–93). Additionally, occasional reports in patients with RE and one patient with FIRES sustain a possible therapeutical application for rituximab in these diseases (94, 95). Natalizumab is a humanized monoclonal antibody targeting α4-integrin (CD49d) on the surface of lymphocytes (96). The interaction between this integrin and the VCAM receptor is involved in the process of diapedesis and hence crucial to lymphocyte extravasation to the CNS. Natalizumab has been prominently employed in patients with multiple sclerosis, with good therapeutic results despite the onset of progressive multifocal leukoencephalopathy (96, 97). The use of natalizumab has been studied in animal models of RE (98) and one patient with RE treated with natalizumab is reported in literature (99).

## Systematic Review of the Literature

### Objective of the Systematic Review

This systematic review aims to assess whether anti-cytokine (anti-IL-1, anti-IL-6, anti-TNF), anti-CD20, and anti-α4-integrin agents could be effective and safe for the treatment of DRE and RSE.

### Methods

#### Protocol

This systematic review was performed following the preferred reporting items for systematic reviews and meta-analysis protocol (PRISMA-P) reporting guidelines (100). The review protocol has not been registered.

#### Inclusion Criteria

##### Population

The study included patients with no age restriction and diagnosed with DRE or RSE. Patients with DRE or RSE caused by specific syndromes (autoimmune encephalitis, LSCS, RE) were included.

##### Intervention

We included patients receiving treatment with anti-IL-1 (anakinra, canakinumab), anti-IL-6 (tocilizumab), anti-TNF (infliximab, adalimumab, etanercept), anti-CD20 (rituximab), anti- anti-α4-integrin (natalizumab) agents.

##### Comparators

Patients receiving ASM, corticosteroids, and other conventional immunosuppressive agents or placebo were included. Studies not including a comparator arm have also been included.

##### Outcomes

The primary outcomes for this review were the efficacy in decreasing or arresting seizures in RSE, controlling the epileptic phenotype in DRE, and the safety of the therapeutic agents in epileptic individuals. The secondary outcome was (when available) the analysis of the motor, behavioral, and cognitive function and recovery after treatment.

##### Study Types

We included all the case reports, case series, retrospective, prospective studies, and clinical trials in which the following criteria were met: (1) original publication; (2) human studies focusing on RSE and/or DRE; (3) at least one enrolled patient with no age restriction; (4) only papers published in English; (5) only papers published between 1 January 2016 and August 2021.

#### Exclusion Criteria

We excluded (1) reviews, book chapters, author's replies ad commentaries; (2) animal experiments; (3) all papers published before 2016; (4) all the studies not meeting the inclusion criteria.

#### Search Strategy and Data Sources

A systematic review of the literature concerning the use of these drugs in patients with epilepsy was conducted in May 2021 and updated in September 2021 on the MEDLINE database (through PubMed). The search terminology was constructed using the name of specific anti-IL-1, anti-IL-6, anti-TNF, anti-CD20, and anti-α4-integrin drugs and the terms “status epilepticus,” “epilepsy,” and “seizure” with the use of Boolean operators [e.g., “(anakinra AND status epilepticus) OR (anakinra AND seizures)”], [“(tocilizumab AND status epilepticus) OR (tocilizumab AND seizures)”], [(anti-tumor necrosis factor-alpha) OR (anti-TNF)) OR (tumor necrosis factor inhibitor)) OR (Infliximab)) OR (Etanercept)) OR (Adalimumab)) AND ((Epilepsy) OR (seizures))], [((rituximab) OR (anti-CD20)) AND ((Epilepsy) OR (seizures))], [(natalizumab) AND ((Epilepsy) OR (seizures))]. For all searches, the respective Medical Subject Headings (MeSH) and Emtree terms were used, if available.

#### Study Records

Three review authors (GC, GD, AM) independently screened the titles and abstracts using the previously described inclusion and exclusion criteria. In this first step, the studies considered for potential inclusion were divided into five categories: anti-IL-1, anti-IL-6, anti-TNF agents, anti-CD20, and anti-α4-integrin. In a second step, the same three review authors (GC, DP, AM) evaluated full-text articles and assessed the eligibility for this systematic review. Any disagreement regarding eligibility was resolved through discussion with a third-party member (AO). Data were extracted using a standardized form on Microsoft Excel (Microsoft Corporation, Seattle, USA). The form was designed by GC, and data were extracted by GC, DP, and AM. Any disagreement regarding data extraction was resolved through discussion with a third-party member (AO). The following data were extracted for each study: first author, publication year, number of enrolled patients and their age, study design, specific drugs used, dosage and route of administration (if available), the timing of administration, patients outcomes (decrease in seizure frequency/arrest of seizures or status epilepticus. motor, behavioral and cognitive outcome, adverse events) and the dosage of serum and CSF cytokines. The data obtained from extraction were synthesized through quantitative and qualitative analysis.

#### Risk of Bias Assessment and Quality of the Evidence

The risk of bias was evaluated using the risk of bias in non-randomized studies of interventions (ROBINS-I) tool (101) for non-randomized studies and the evidence-based medicine (EBM) indications (102) for case series and reports. Each study was assessed by one review author (GC). To estimate the level of evidence reached by this work, the grading of recommendations, assessment, development, and evaluation (GRADE) (103) approach was used. The risk of bias, precision, consistency, directness, and publication bias was analyzed.

### Systematic Literature Review: Anti-IL-1 Agents and Epilepsy

#### Study Selection

**Figure 3:**
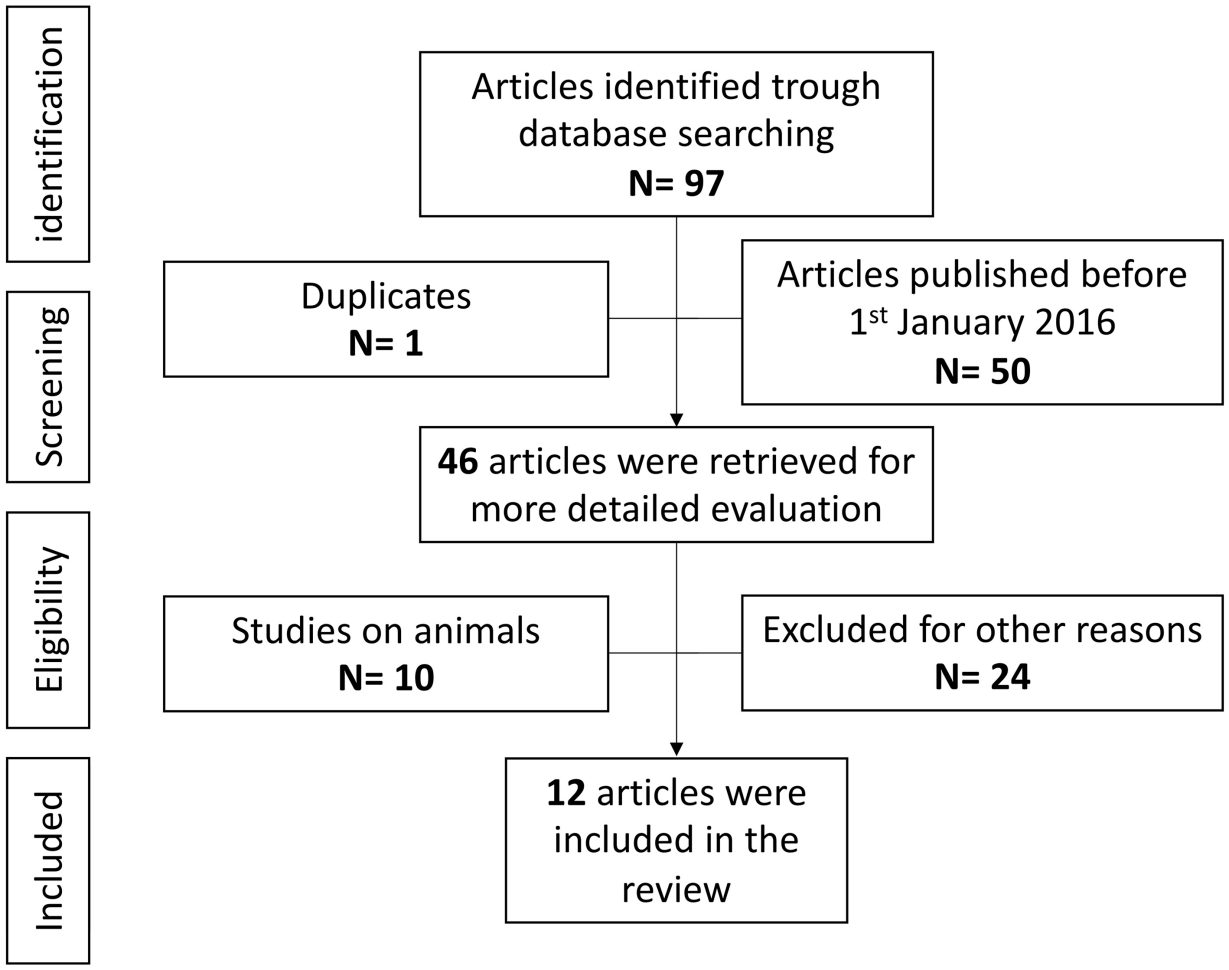
Anti-IL-1 agents and epilepsy, literature review.

Overall, 97 references were identified using the search strategy. After identifying one duplicate and removing 50 articles published before 1 January 2016, the full text of 46 articles was retrieved and evaluated in detail. After a full-text assessment 34 articles were excluded: 10 studies were on animals and 24 were not relevant to the topic of the present review. One paper on multifocal neutrophilic meningoencephalitis (MNM) was excluded as did not directly analyze the effect on the epileptic phenotype (104). Twelve articles were finally included in this review (Figure 3), for a total of 37 cases of epilepsy with different etiologies (105–116). All publications were in English, six were from the USA, three were from Europe, one from Asia, and two were international studies, involving different countries. Eleven publications were case reports, while the last was a retrospective observational study.

#### Results

Among the 37 patients included in this systematic review, the median age was 7 years. Thirty-two patients suffered from FIRES; the other patients suffered from new-onset refractory status epilepticus (NORSE) (*n* = 1), super-refractory status epilepticus (SRSE) (*n* = 1), RE (*n* = 1), encephalopathy with electrical status epilepticus in sleep (ESES) (*n* = 1), and DRE (*n* = 1) (Tables 1, 2).

**Table 2:** Systematic review of anti-IL-1 agents in epilepsy.

**References**	**Disease**	***N*, (age)**	**Study design**	**Intervention**	**Timing**	**Efficacy**	**Safety**
Sa et al. (105)	FIRES	2 Pt 1: 1.9 years Pt 2. 2.5 years	Case Report	Pt 1 Anakinra 5 mg/Kg/day s.c for 14 days	Start: Day 43	From Day 51 seizures decreased in frequency and on Day 60 these stopped. After 3 weeks new onset of seizures (2–5/month)	No adverse effects
				Pt 2 Anakinra 10 mg/Kg/day s.c for 90 days	Start: day 22 Discontinued after 3 months of treatment	No improvement	No adverse effects
Yang et al. (106)	FIRES	1 (6 years old)	Case Report	Anakinka 100 mg s.c. twice daily for 1 year	Start: Day 28	Resolution of seizures after 4 days. Stop ketogenic diet after 9 months. Follow-up: 1 seizure/month	No adverse effects
Kern-Smith et al. (107)	NORSE	1 (5 years old)	Case Report	Anakinra for 13 days (posology not specified)	Start: Day 12 Stop: Day 24	Stop midazolam infusion, without return of electrographic status epilepticus, after 2 days	No adverse effects
Dilena et al. (108)	FIRES	1 (10 years old)	Case Report	Anankinra 2.5 mg/kg/day (100 mg) s.c and, 3 days after, 2.5 mg/kg twice daily	Start: after 18 months from diagnosis Stop: 7 months after	Full seizure control after 3 days	No adverse effects
Jyonouchi and Geng (109)	ESES	1 (6 years old)	Case Report	Anakinra 100 mg/day s.c.	Start: 25 months after diagnosis	Despite the improvement of behavioral symptoms, ESES pattern persisted.	No adverse effects
Lai et al. (110)	FIRES	25, (5–11 years old)	Retrospective	Anakinra 3–5 mg/kg/day (initial dose) Anakinra 4–9 mg/kg/day (final dose)	Start: 20 days after the onset of FIRES Stop: 86 days after	Earlier anakinra initiation after seizure onset was associated with shorter duration of mechanical ventilation, and ICU and hospital LOS. Amongst children with available seizure frequency data, 11/15 (73.3%) exhibited> 50% seizure reduction at 1 week of anakinra treatment. 3/25 (12%) died	3/25 (12%) developed DRESS 2/25 (8%) developed cytopenia 10/25 (40%) developed infections (1 discontinued anakinra for infections)
Westbrook et al. (111)	FIRES	1 (21 years old)	Case Report	Anakinra 100 mg 3 times daily s.c (Initial dose) Anakinra 100 mg twice daily s.c (after 10 days) Anakinra 100 mg once daily s.c. (after 25 days)	Start: 32 days after the diagnosis of FIRES Stop: 1 year after at which time discontinuation will be discussed	Full seizure control after 24 h	No adverse effects
Kenney- Jung et al. (112)	FIRES	1 (32 months old)	Case Report	Anakinra 5 mg/kg/twice daily s.c. Anakinra 5 mg/kg/twice daily s.c.	2 cycles: Day 6–23 Day 54-ongoing	Improved seizure control in both cycles (from 5.8 to 1.3 seizure/day in the first cycle; from 8 to 0.17 seizure/day in the second). Twelve months after initial presentation, the patient experiences rare focal seizures.	Development of DRESS (day 22, followed by discontinuation)
DeSena et al. (113)	DRE	1 (14 years old)	Case Report	Anakinra 100 mg daily Anakinra 100 mg twice daily Canakinumab 300 mg every 4 weeks	Start: 2 years Stop: After 4 weeks After: 2 months	Rapid ~80% reduction in seizure frequency (from 4 to 15/day to 4/week). No clinically evident seizures. Improvements in her fatigue, general malaise, quality of life, and academic performance. Long periods of being seizure-free, currently averaging one seizure per several months.	No adverse effects
Stredny et al. (114)	FIRES	1 (6 years old)	Case Report	Anakinra 20 mg/kg daily	From day 6 to 20 of hospitalization	No clinical response	No adverse effects
Mochol et al. (115)	RE	1 (43 years old)	Case Report	Anakinra 100 mg daily sc	26 years after disease presentation. 1st cycle: 2 months 2nd cycle: 7 months	Complete seizure control after 1 week of treatment. Relapse after 2 weeks of withdrawal 2nd cycle: full seizure control after 10 days 13 months seizure-free	Pneumonia
Choi et al. (116)	SRSE in AE	1 (38 years old)	Case Report	Anakinra 100 mg daily s.c	From week 12 Duration: 12 days	Resolution of status epilepticus Recovery in communication and walking)	No adverse effects

*AE, autoimmune encephalitis; DRE, drug-resistant epilepsy; DRESS, Drug rash with eosinophilia and systemic symptoms; ESES, Encephalopathy with electrical status epilepticus in sleep; FIRES, Febrile infection-related epilepsy syndrome; NORSE, New-onset refractory status epilepticus; RE, Rasmussen encephalitis; SRSE, super-refractory status epilepticus*.

Anakinra was administered with various posologies, ranging from 3 to 20 mg/Kg/day, with a maximum dose of 100 mg SC per administration; canakinumab 300 mg SC was used in one study; no studies were reporting the use of rilonacept. Anti-IL-1 drugs were administrated at least as third-line therapy, after ASM (*n* = 37), corticosteroids (*n* = 32), immunosuppressive agents (sirolimus, *n* = 1), ketogenic diet (*n* = 23), intravenous immunoglobulins (*n* = 28), rituximab (*n* = 7), or plasma exchange (*n* = 14). Two (=2) patients underwent deep brain stimulation and one underwent surgery for DRE. Regarding the timing of administration, anti-IL-1 was started early in 32 cases, with a median delay of 20 days from the onset of seizures. In the remaining five cases, anti-IL-1 treatment was administrated after more than 4 months. Except for five cases in which there weren't improvements (three of them died after drug discontinuation) (105, 110, 114), the use of anti-IL-1 was effective in reducing the seizure burden. Indeed, its use significantly reduced seizures (>50%) in 11/15 of the patients described in the cohort by Lai et al. with available clinical data (110), and in 9/12 of the patients described in case reports (Table 2). Concerning safety issues, the most frequently reported adverse effect was the development of infections, which have been reported in 11 patients, while four patients experienced drug rash with eosinophilia and systemic symptoms (DRESS) (110, 112). Regarding the cognitive, motor, and behavioral recovery, although in most of the patients presented in case reports (9/12) an improvement was reported, the outcome has been mostly described qualitatively. The study by Lai et al. evidenced that, among the 22 surviving patients, 50% had motor deficit, 77% attention deficit, and more than 50% of the patients had speech, memory, or executive function deficit. In this study, 45% of the surviving patients had mild or moderate disability measured using the pediatric cerebral performance category (PCPC) scale, and 22,7% had a severe disability or vegetative state. Additionally, all the surviving patients in this study have DRE at last follow-up (110). The cytokine serum and CSF levels are reported in 13/37 (35%) and 14/37(37,8%) of the patients, respectively. In the analysis of patients treated with anti-IL drugs, we evidenced some potential biases deriving from missing data, as seizure frequency was not reported for all the included patients (particularly, data are not available for 10/25 patients in the cohort by Lai et al.) (110). Additionally, the lack of use of specific assessment scales did not allow a deeper evaluation of the motor, behavioral and cognitive outcome.

### Systematic Literature Review: Anti-IL-6 Agents and Epilepsy

#### Study Selection

**Figure 4:**
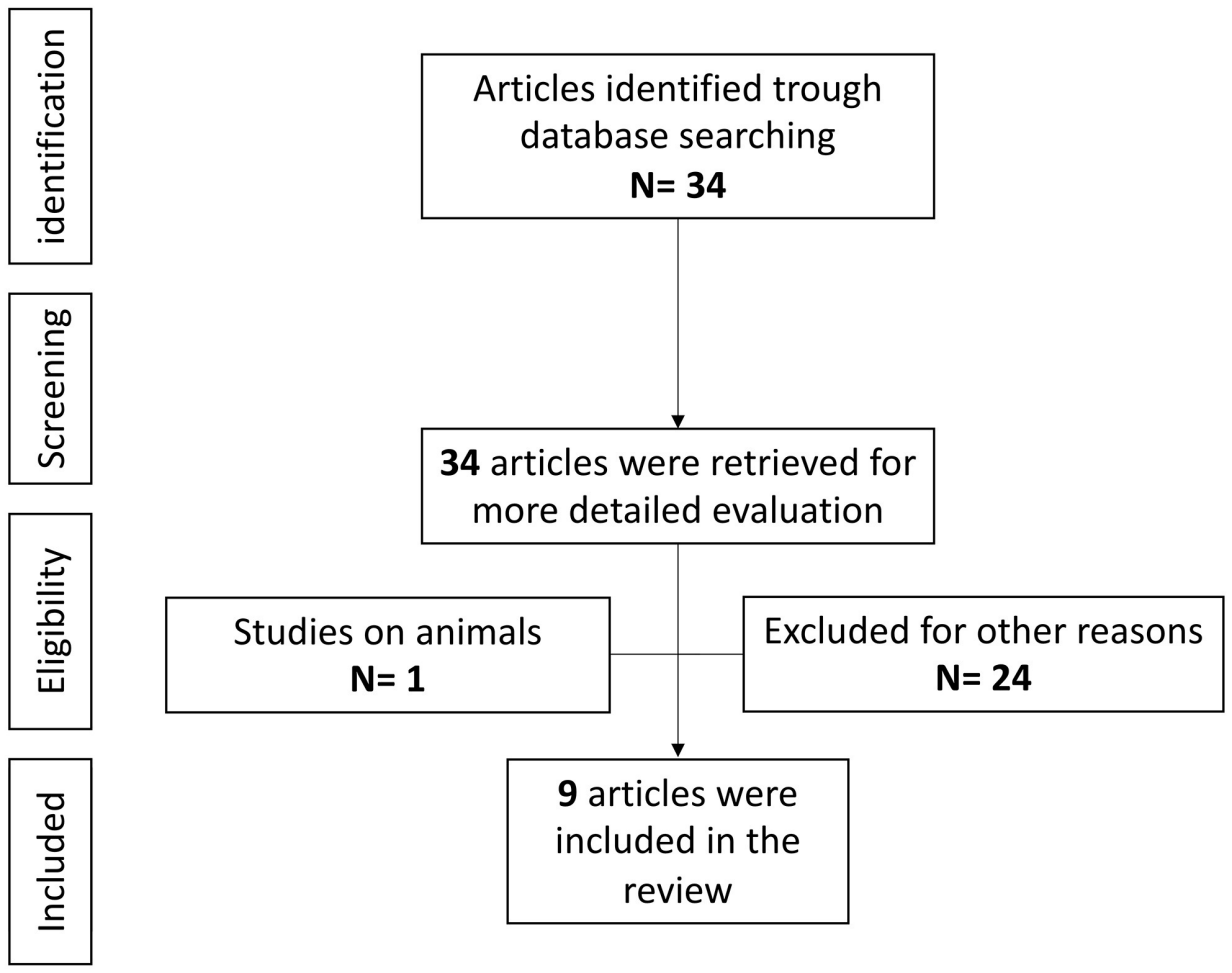
Anti-IL-6 agents and epilepsy, literature review.

Overall, 32 references were identified using the search strategy. The full text of these 34 articles was retrieved and evaluated in detail, and 25 articles were excluded based on the exclusion criteria. Nine articles were finally included in this review (Figure 4), for a total of 16 patients with epilepsy with different etiologies (114, 117–124). Two studies, performed on patients with autoimmune encephalitis and anti-N-methyl-D-aspartate receptor (NMDAR) encephalitis, were excluded as epilepsy was not considered as a specific outcome of the study, although the studies evidenced a global disease activity improvement after treatment with tocilizumab (125, 126). All publications were in English, three from the USA, four from Europe, and two from Asia. Eight publications were case reports, another (*n* = 1), was a prospective study.

#### Results

Among the 16 patients included in this systematic review, the median age was 23 years. Patients suffered from NORSE (*n* = 10), SRSE (*n* = 2), DRE associated with LSCS (*n* = 2), limbic encephalitis with anti-CASPR2 antibodies (*n* = 1), and FIRES (*n* = 1) (Table 3). Tocilizumab was administered in all 16 cases either SC or IV. No other anti-IL-6 was used. Tocilizumab was administered at least as a third-line therapy, after ASM (*n* = 16), corticosteroids (*n* = 14), immunosuppressive agents (*n* = 2), ketogenic diet (*n* = 3), intravenous immunoglobulins (*n* = 14), plasma exchange (*n* = 5), or others (anakinra *n* = 1, electroconvulsive therapy *n* = 1). Tocilizumab was started within the first 6 months of illness in 15 cases (median: 25 days). In only one patient tocilizumab was administered after more than 12 months from the disease onset (Table 3). From the available data, it emerges that the use of anti-IL-6 was effective for partial or complete seizure control in most patients [6/7 of the patients described in the cohort study by Jun et al. (124), 9/9 of the patients described in case reports showed reduction or arrest of the seizures]. Concerning safety, although tocilizumab has been well-tolerated by the majority of the patients, our analysis evidenced three cases of infection (one pneumonia and two cases of sepsis), and two patients developed leukopenia (123, 124). The motor, behavioral, and cognitive outcome has been reported mostly with qualitative assessment, showing a reported improvement in seven out of the nine single case reports, with the persistence of behavioral dysregulation (114), mild cognitive impairment, and mild ataxia (118) in two patients. In the study by Jun et al. and the case presented by Benucci et al. the modified Rankin scale (mRS) was used, demonstrating an improvement at follow-up in 4/7 survived patients (122, 124). The dosage of serum and CSF cytokines was available each in 10/16 (62,5%) of the patients. Also in the study of patients treated with tocilizumab, the risk of biases (particularly, missing data) is present, with data being often presented only with qualitative assessment. Additionally, the high rate of therapeutic response in case reports suggests the possibility of a publication bias.

**Table 3:** Systematic review of anti-IL-6 agents in epilepsy.

**References**	**Disease**	**PTS *N* Age**	**Study design**	**Intervention**	**Timing**	**Efficacy**	**Safety**
Magro et al. (117)	CNS disease and DRE associated with LSCS.	1 (22 years old)	Case Report	Tocilizumab 162 mg S.C. once a week	Start: 6 months after	Noticeable improvement in cognitive and affective symptoms with decrease in seizure frequency. Resolution of many of the enhancing lesions on brain MRI	No adverse effects
Stredny et al. (114)	FIRES	1 (6 years old)	Case Report	Tocilizumab 12 mg/kg S.C. every 2 weeks	Start: Day 20 Stop: Day 76	Reduction of seizure	No adverse effects
Donnelly et al. (118)	NORSE	1 (26 years old)	Case Report	Tocilizumab 300 mg IV. for two times	First dose: 9 weeks after the beginning of treatment Second dose: 12 weeks after the beginning of treatment	Stop seizures after 48 h	No adverse effects
Osminina et al. (119)	CNS disease and DRE associated with LSCS.	1 (2 years 10 months)	Case Report	Tocilizumab 10 mg/kg IV. once in 4 weeks	Start: 16 months after the beginning of symptoms Stop: 26 months after first infusion of Tz.	Reduction of periventricular focus; stop seizures.	No adverse effects
Jaafar et al. (120)	SRSE	1 (8 years old)	Case Report	Tocilizumab, 8 mg/kg/day S.C. divided in two doses 1 week apart	Start: 10 days after admission to hospital	Stops seizure 24 h after	No adverse effects
Cantarín-Extremera et al. (121)	NORSE	2 Pt 1: 1.9 years old Pt 2: 2.7 years old	Case Report	Pt 1 Tocilizumab 4 mg/kg once a week	Start: Day 21 Stop: Day 28	Seizures decrease in frequency, in VEEG critical patterns had disappeared.	No adverse effects
				Pt 2 Tocilizumab 4 mg/kg for 2 times	Start: Day 30 and 40	48–72 h after the first dose, the seizures began to decrease progressively in frequency and intensity, there was global neurological improvement, recovering normality in terms of language, level of consciousness, and motor capacity, but persisting hyperactivity.	No adverse effects
Jun et al. (124)	NORSE	7 [median 25 years old (22–64)]	Prospective	Tocilizumab 4 mg/kg for 2 cycles in 1-week intervals, a monthly dose (8 mg/kg) was added if needed	Start: Median day 25 (6–73)	Resolution of status epilepticus in 6/7 patients 3/6 of the survived patients showed improvement on the mRS	2/7 (2.9%) leukopenia 1/7 (1.4%) diarrhea 1/7 (1.4%) pneumonia 1/7 (1.4%) sepsis
Benucci et al. (122)	Limbic Encephalitis with Anti-CASPR2 Antibodies	1 (64 years old)	Case Report	Tocilizumab 8 mg/kg IV. once a month for 6 months, then 162 mg every week s.c	Start: 2–3 months after admission.	Full seizure control Resolution of behavioral changes and seizures Improvement in mRS	No adverse effects
Vallecoccia et al. (123)	SRSE	1 (34 years old)	Case Report	2 doses of tocilizumab 4 mg/kg at a 1-week interval	Start: day 24	Partial recovery after 7–10 days from the first administration. Resolution of the clinical picture after 1 month (*ketamine and ketogenic diet added)	Sepsis by drug-resistant pathogen

*CNS, Central nervous system; DRE: drug-resistant epilepsy; FIRES, Febrile infection-related epilepsy syndrome; LSCS, Linear scleroderma “en coup de sabre;” mRS, modified Rankin scale; NORSE, New-onset refractory status epilepticus; RSE, refractory status epilepticus; SRSE, Super-refractory status epilepticus*.

### Systematic Literature Review: Anti-TNFα Agents and Epilepsy

#### Study Selection

Overall a total of 142 references were identified during the initial electronic search, nine of which were marked as not eligible by automation tools because they were not published in English language.

**Figure 5:**
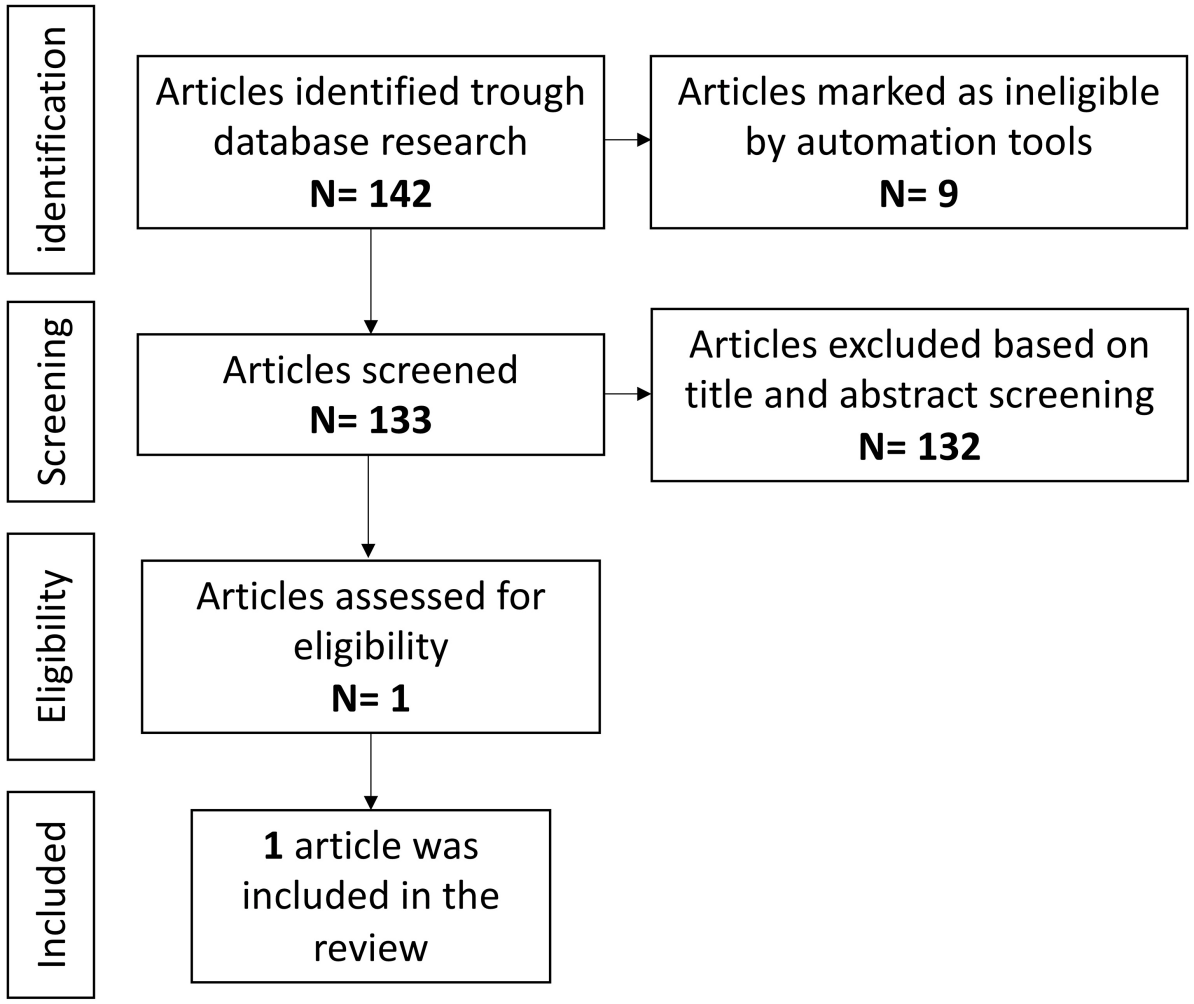
Anti-TNF agents and epilepsy, literature review.

A total of 133 potentially eligible studies were selected. Among these, 132 were excluded (Figure 5). One study resulted eligible and was hence included in this review: a multicenter, open-label, prospective study (127).

#### Results

A total of 11 patients with RE were included in the study (Table 4). The median age at first seizure was 6.5 years (range 1.5–37 years). All patients received adalimumab SC (24 mg/m^2^ with a maximum of 40 mg) every 14 days. Before adalimumab, patients received treatment with corticosteroids (*n* = 11), in addition to immunoglobulins (*n* = 8) and azathioprine (*n* = 1). The Anti TNF-α agent was started with a median delay of 31 months after the first seizure (range 1 month-16 years). The primary outcome was the decrease of seizures frequency, considering “responders” patients experiencing a decrease in seizure frequency by at least 50%. The secondary outcome measures were neurologic and cognitive outcomes and side effects of the treatment. Although none of the patients became seizure-free, five patients were considered as responders and another one experienced a transitory improvement in frequency of seizures. In three of the five patients, a stabilization of cognitive decline was observed and two patients had improvement of their motor deficiencies. According to the authors, the response to the treatment seemed to occur more likely in slowly progressive forms of RE and patients with concomitant autoimmune diseases (uveitis and juvenile arthritis). None of the responders underwent hemispherectomy, considering the absence of a severe motor and cognitive deficit. On the other hand, three patients experienced a severe progression of the disease, with the requirement of hemispherectomy. Concerning safety, in 1 patient adalimumab was discontinued due to an elevation of creatine kinase levels and another patient showed a superficial skin infection that did not require interruption of the treatment and was successfully controlled with a local antiseptic (127).

**Table 4:** Systematic review of anti-TNF agents in epilepsy.

**References**	**Disease**	**PTS *N* Age**	**Study design**	**Intervention**	**Timing**	**Efficacy**	**Safety**
Lagarde et al. (127)	Rasmussen encephalitis.	11 [median 6.5 years (1.5–37)]	Multicenter, open-label, prospective study	Adalimumab 24 mg/m^2^ s.c. (maximum 40 mg), every 14 days	Start: Median delay 31 months (range 1–192) after diagnosis	Complete response (>50% seizure frequency decrease) in 5 patients; 3 of these 5 patients had stabilization of functional deficits. One patient showed significant but transitory (6 months) improvement.	One patient discontinued adalimumab due to mild increase of creatine kinase blood levels (normalized after 3 weeks); one patient showed superficial skin infection (not requiring discontinuation of treatment).

### Systematic Literature Review: Anti-CD20 Agents and Epilepsy

#### Study Selection

**Figure 6:**
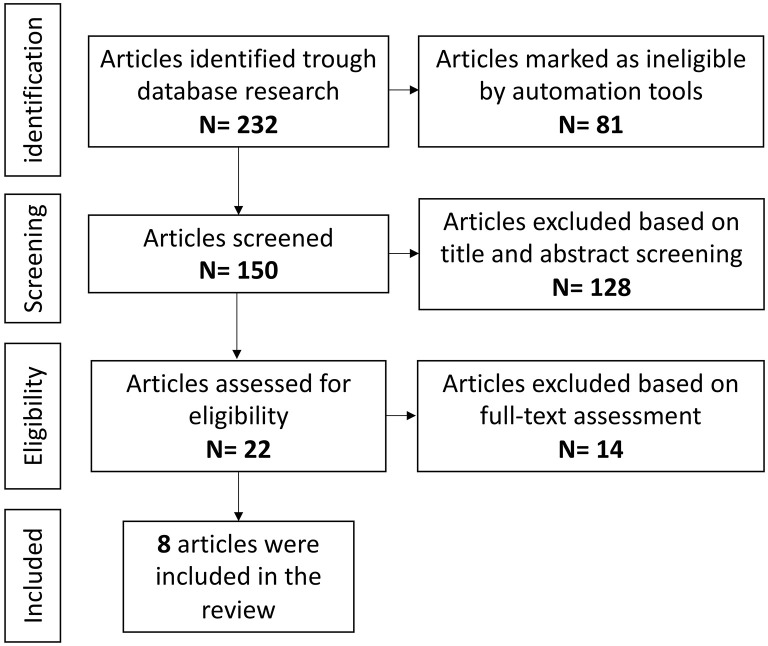
Anti-CD20 agents and epilepsy, literature review.

Overall a total of 232 references were identified during the initial electronic search, 82 of which were marked as not eligible by automation tools because published before 1 January 2016 and/or not in English language. A total of 150 potentially eligible studies were selected. Among these, 128 were excluded for irrelevancy after a screening of the titles and abstracts (Figure 6). After a full-text assessment of the remaining 22 studies, eight of them resulted eligible and were therefore included in this review (124, 128–134). Six publications were case reports and two were prospective studies.

#### Results

A total of 26 patients with different epileptic disorders were included in this review (Table 5). The median age of the patients was 32 years (range 11–72). The patients suffered from RE (*n* = 3) and from AE due to anti-NMDAR (*n* = 11), anti-LGI1 (*n* = 5), anti-voltage-gated calcium channels (VGCC) antibodies (*n* = 1), or onconeural antibodies (anti-Ma2/Ta) (*n* = 1). Four patients presented a cryptogenic RSE. The posology of rituximab was variable within the different cases and in one case it was not specified (Table 5). For all patients, rituximab was started as second-line immunotherapy after the administration of IVIg and steroids. The timing of administration was variable and, given the heterogeneity of the diseases, it is difficult to determine homogeneous timepoints of reference for all the patients. Overall, the treatment with rituximab was effective in abolishing or significantly reducing the burden of seizures in 16/26 patients (61,5%), while in 10/26 patients, the therapy was ineffective. One patient with RE underwent functional hemispherectomy with remission of seizures (132). As discussed in Section Results, the six patients from the cohort of Jun et al. received Tocilizumab with the resolution of status epilepticus (124). Of the three patients in the study of Byun et al. that did not show a clinical change in the course of their disease after the administration of rituximab, one deceased for reasons not related to the therapy itself (128). The two others continued to show seizures and there is no mention of additional treatments. Data concerning the behavioral, cognitive, and motor outcomes were available for seven patients (130–134). An improvement was reported in six patients and in five of them the behavioral and cognitive functions were restored to premorbid condition. One patient with RE, albeit improved, showed residual motor and speech dysfunctions (130). In another case, the administration of rituximab resulted ineffective. Finally, only one patient had to discontinue the therapy due to a cutaneous rash associated with pruritus that appeared 24 h after the administration (128). Two other patients experienced mild infusion-related reactions and were able to continue the therapy (126). No adverse effects were reported in the other patients.

**Table 5:** Systematic review of anti-CD20 agents in epilepsy.

**References**	**Disease**	***N*, (age)**	**Study design**	**Intervention**	**Timing**	**Efficacy**	**Safety**
Cheli et al. (134)	Anti-LGI1 encephalitis with DRE	1 (54 years old)	Case Report	Rituximab 1,000 mg/day IV, 2 doses 15 days apart then one single dose after 6 months.	Start: 7 weeks after the onset of seizures. Stop: 9 weeks after the onset of seizures.	No further seizures occurred after the treatment. The neuropsychological evaluation resulted within normal range. After 6 months, the patient experienced a cognitive relapse that resolved after the administration of a single dose of Rituximab.	No adverse effects
Kurukumbi et al. (133)	Patient 1: anti-NMDR encephalitis with RSE Patient 2: anti- LGI1 encephalitis with DRE Patient 3: N-type anti-VGCC encephalitis with RSE	3 Pt 1: 32 years Pt 2: 72 years Pt 3: 19 years	Case Series	Pt 1: Rituximab 375 mg/m^2^/day IV weekly for 4 weeks, then rituximab 1,000 mg IV every 6 months Pt 2: Rituximab 1,000 mg IV every 6 months Pt 3: Rituximab 1,000 mg IV every 6 months	Pt 1, start: 27 days after the onset of seizures. Pt 2, start: 12 months after diagnosis Pt 3, start: 5 days after the onset of seizures.	Pt 1: resolution of seizures and behavioral disorders, with a return to baseline cognition and personality Pt 2: electrographic and clinical seizure freedom with return of premorbid cognitive function Pt 3: abrogation of seizures and return to baseline functioning	No adverse effects
Sansevere et al. (132)	RE	1 (11 years old)	Case Report	Rituximab 375 mg/m^2^ weekly for 4 weeks	Start: 5 days after diagnosis Stop: 33 days after diagnosis	No clinical response. Functional hemispherectomy with right hemisphere deafferentation was performed 3 months after the final dose of rituximab	No adverse effects
Schneider et al. (131)	Anti-NMDAR encephalitis with RSE	1 (22 years old)	Case Report	Rituximab 500 mg IV, followed by a second dose after 6 months and a third after 16 months	Start: not specified Stop: 16 months after the first administration	Complete remission of epileptic seizures and psychotic symptoms	No adverse effects
Jun et al. (124)	NORSE^*^	6 [median 36 years (22–61)]	Prospective	Rituximab 375 mg/m^2^/day IV weekly	Not specified	Persistence of SE despite the treatment. Patients eventually received Tocilizumab	No adverse effects
El Tawil et al. (130)	RE with DRE	1 (61 years old)	Case Report	Rituximab (posology not specified)	Start: 10 years after diagnosis Stop: not specified	Clear and sustained improvement in seizure frequency and severity and patient's disabilities	No adverse effects
Timarova et al. (129)	RE with RSE	1 (32 years old)	Case Report	Rituximab 375 mg/m^2^/day IV weekly (two cycles 22 months apart)	Start: not specified Stop: 22 months after the first administration	Reduction of epileptic seizures with residual 2–3 partial seizures per week. Worsening 18 months after (partial seizures rose to 6 per day). After the second cycle, persistence of one partial seizure per day.	No adverse effects
Byun et al. (128)	AE^**^	12 [median 32 years (18–68)]	Prospective study	Rituximab 375 mg/m^2^/day IV weekly for 4 weeks. The treatment was then repeated every month.	Start: 3–9 weeks after first immunotherapy cycle (with steroid or IVIg).	Remission of seizures in 8/12 patients at 6 months; seizures reduction > 50% in 1/12 patient; no clinical change in 3/12 patients.	Infusion-related reactions (headache, dizziness, chest discomfort) (*n* = 2); rash with pruritus 24 h after the infusion (*n* = 1). This patient discontinued the treatment.

*AE, autoimmune encephalitis; DRE, drug-resistant epilepsy; IV, intravenous; IVIg, intravenous immunoglobulins; LGI1, leucine rich glioma inactivated 1; NMDAR, N-methyl-D-aspartate receptor; NORSE, New-onset refractory status epilepticus; RE, Rasmussen encephalitis; SRSE, super-refractory status epilepticus; VGCC, voltage-gated calcium channel*.

* *One patient suffered from anti-NMDAR encephalitis, in the other cases the disease was cryptogenic*.

** *AE in these patients was due to anti-NMDAR (n = 8), anti-LGI1 (n = 3), and anti-Ma2/Ta (n = 1)*.

### Systematic Literature Review: Anti-α4-Integrin Agents and Epilepsy

#### Study Selection

**Figure 7:**
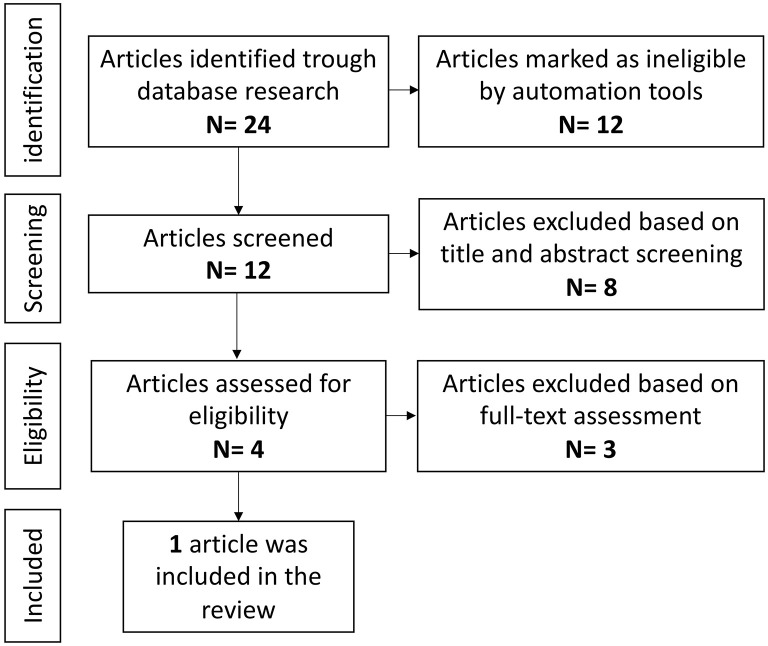
Anti-α4-integrin agents and epilepsy, literature review.

Overall a total of 24 references were identified during the initial electronic search, 12 of which were marked as not eligible by automation tools because published before 1 January 2016 and/or not in English language. A total of 12 potentially eligible studies were selected. Among these, eight were excluded for irrelevancy after a screening of the titles and abstracts (Figure 7). After a full-text assessment of the remaining four studies, three of them resulted ineligible, since the evaluation of seizures frequency and/or severity was not in the outcomes of the studies, or because studies were not concerning patients with RSE or DRE. Therefore, one single study was included in this review, a randomized, placebo-controlled, double-blinded study (phase 2 study OPUS, NCT03283371) (135).

#### Results

A total of 32 patients with drug-resistant focal epilepsy were included in this review (Table 6). The mean age was 42.8 years (±14.56). All patients received natalizumab 300 mg IV every 4 weeks for 24 weeks. The timing of administration was not specified. The primary endpoint of efficacy of the study included was to evaluate the change from baseline in seizure frequency (number of seizures per 28 days) from weeks 8 to 24 of the placebo-controlled period. Overall, the natalizumab-treated group showed a greater reduction in seizure frequency compared to placebo (−14.4%), although the predefined threshold for therapeutic success of 31% relative reduction was not achieved (135). Concerning the secondary endpoints, despite a reduction of ≥50% in seizure frequency from baseline during weeks 8–24 of treatment in 10/32 participants (31.3% compared to 17.6% of the placebo group), none of the participants that received natalizumab remained free from seizures. Reportedly, one patient experienced an inadequate treatment response and withdrew from the treatment (135). Finally, the exploratory endpoints included change from baseline in frequencies of focal to bilateral tonic-clonic seizures and focal seizures. Participants of the natalizumab-treated group showed respectively a decrease of 21.34% and an increase of 6.61% in frequency over placebo. The endpoints of the study did not pertain to the motor, behavioral or cognitive outcome of the participants. Concerning safety, although adverse events were reported in 24/32 participants, ranging from mild (15/24) to moderate (8/15) and severe (1/32), only 2/32 showed events of special interest and only one of these participants discontinued the treatment due to urticaria (135).

**Table 6:** Systematic review of anti-α4-integrin agents in epilepsy.

**References**	**Disease**	***N*, (age)**	**Study design**	**Intervention**	**Timing**	**Efficacy**	**Safety**
French et al. (135)	DRE	32 [mean 42.8 years (±14.56)]	Randomized, placebo-controlled, double-blinded study	Natalizumab 300 mg IV every 4 weeks for 24 weeks	Start: not specified Stop: 24 weeks after the first dose.	Compared to placebo, the natalizumab-treated group showed a greater reduction in seizure frequency from baseline. 10/32 participants showed a reduction of ≥50% from baseline in seizure frequency during weeks 8–24. None of the participants remained free from seizures. One participant experienced an inadequate treatment response (e.g., did not modify ASMs after week 12 of the placebo-controlled period or did not discontinue the treatment after the active run-in period due to lack of efficacy)	24/32 participants experienced adverse effects ranging from mild (15/24), moderate (8/15) and severe (1/32). One participant experienced a serious event (urticaria) and discontinued the treatment.

*ASM, anti-seizure medication; DRE, drug-resistant epilepsy; IV, intravenous*.

### Strengths and Limitations

**Figure 8:**
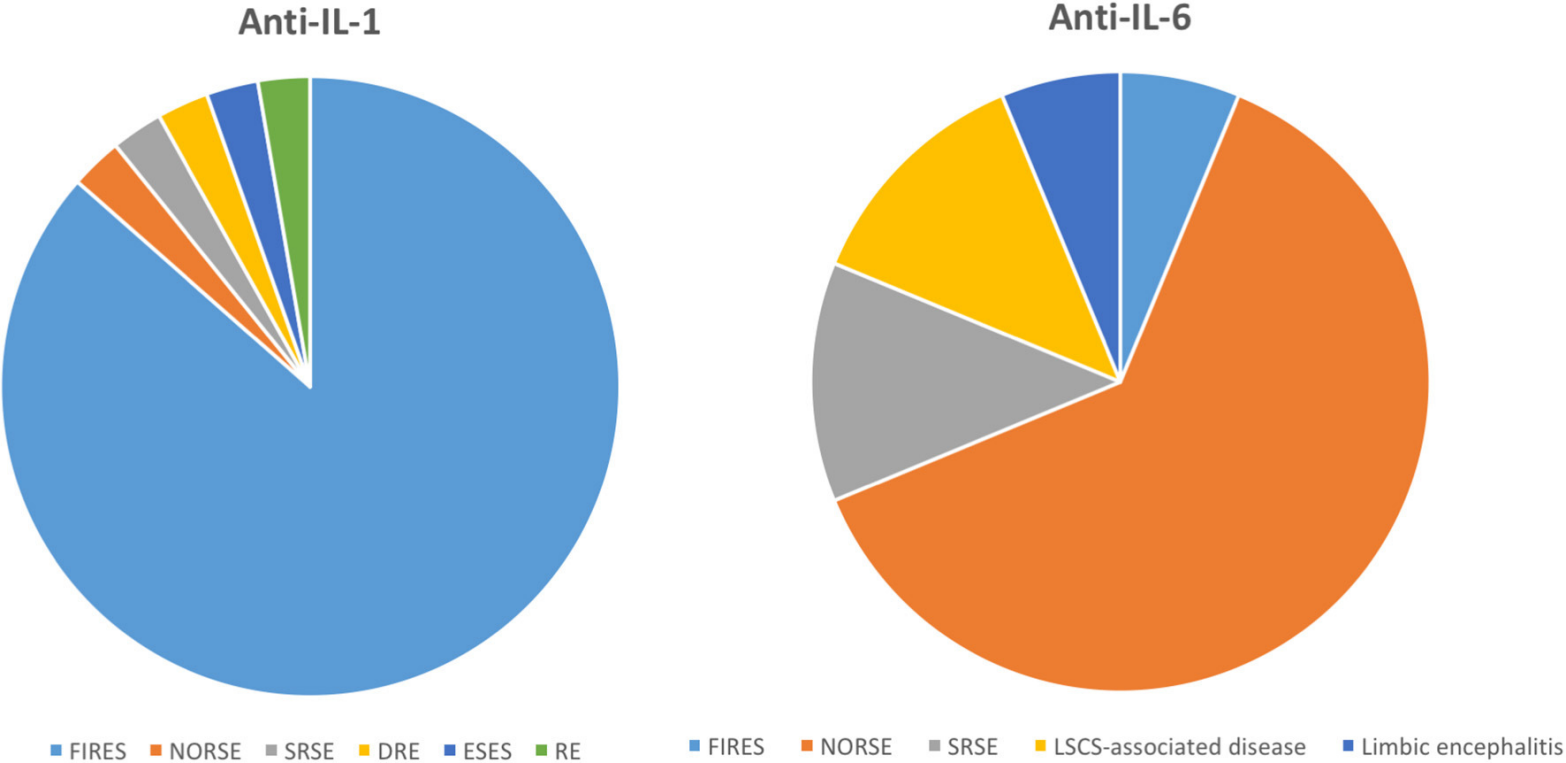
Use of anakinra and tocilizumab in epilepsy, data from the literature.

This is the first systematic review exploring the use of anti-cytokine agents and anti-lymphocyte agents in individuals with DRE or RSE. This study has different limitations. Firstly, data on the use of anti-cytokine agents are mostly derived from isolated case reports and case series, with no randomized clinical trials, and the age range of the included patients is considerable. This significantly raises the risk of publication bias, thus favoring the publication of positive reports. Moreover, the administration of concomitant treatments (ASMs, corticosteroids, other immunosuppressive agents) can represent a considerable confounding factor. Therefore, the level of evidence is currently low. However, it is worth highlighting that the administration of anti-IL-1 agents (mainly anakinra) and tocilizumab in patients with severe DRE, such as RSE or FIRES, has shown a reduced seizure burden in most of the described patients, and a good safety profile is usually well-tolerated. Particularly, literature data report that more than 50% of the patients with FIRES respond to anakinra, while tocilizumab has been mostly used in patients with NORSE and SRSE showing partial or complete seizure control in almost all the described cases (Figure 8). Therefore, despite the mentioned limitations of this work, literature data suggest considering anti-IL-1 or anti-IL-6 therapeutic approaches in these conditions. Data on the use of adalimumab in patients with RE encourage further studies to assess whether this drug or other TNF-inhibitors could be effective in the treatment of those forms of RE refractory to other immunotherapies, but without the criteria for a surgical approach. Selected patients, like those with slowly progressive forms, may benefit from treatment with Anti TNF-α agents, while less is known regarding their use in other forms of epilepsy since the literature is scarce and studies on animal models are limited. Apart from the effect on seizures, anti-cytokine agents could significantly affect also motor, behavioral, and cognitive recovery, although this outcome has been heterogeneously reported in the different studies, with only four studies analyzing specific scales (110, 122, 124, 127). Notably, the serum and CSF cytokine profile was determined in <50% of the included patients. Although this investigation is not part of routine clinical practice (difficulty of interpretation, limited availability) the increasing knowledge of the involvement of cytokines in DRE and RSE could lead to a more diffuse use of this dosage, to provide a therapeutic strategy targeted on the main mediator involved in the individual patient.

The administration of Rituximab resulted to be effective in the majority of patients with DRE and RSE, with a more conspicuous response in individuals affected by AE-related seizures. Once more, a possible limitation and source of bias comes from the fact that studies concerning the use of Rituximab consit mainly in case reports or case series. This may also be the cause of the considerable heterogeneity in the timing and posology of the treatment that was evidenced. Future studies in this field should prioritize the definition of an appropriate treatment regimen.

Although targeting the leukocyte extravasation to brain parenchyma appeared to be a creditable therapeutic opportunity for patients with intractable epilepsy, the results of a randomized, double-blinded, placebo-controlled study showed that the administration of natalizumab did not significantly change seizure frequency in adults with drug-resistant focal epilepsy (135). Future trials, with a larger sample size, may overcome the limits of this latter study in detecting statistically significant differences.

## Other Inflammatory Targets and Epilepsy: Future Therapeutic Perspectives

Preclinical research is focusing on the identification of novel therapeutic targets for the treatment of neuroinflammation. In this regard, the role of agents targeting the human high-mobility group box-1 (HMGB-1) and chemokines are of particular interest. HMGB-1, a regulator of gene transcription and DNA remodeling/repair (136), mediates inflammatory responses *via* interactions with the receptor for advanced glycation end products (RAGE) and toll-like receptor (TLR) 4. It promotes the release of pro-inflammatory cytokines (e.g., TNF-α and IL-6) and acts as a key initiator of inflammation particularly within the brain (136–138). Anti-HMGB1 mAbs have proven to be effective in different mouse models of epilepsy (138–140), reducing also the chronic inflammatory pathways (upregulation of inflammation-related genes, microglial activation, and neuronal cell death). Chemokines are involved at different levels in CNS homeostasis, and have been implicated in the pathogenesis of different CNS diseases, including epilepsy (26, 141). In animal models of epilepsy, the administration of a CCL2 transcription inhibitor (Bindarit) or a selective antagonist of the CCR2 receptor (RS102895) suppressed the LPS-induced seizure enhancement (142, 143). Additionally, CX3CL1/CX3CR1 and the CC-chemokine receptor CCR5, widely expressed in the CNS microglia, have been implicated in the pathogenesis of epilepsy and represent other potential therapeutic targets (144–147). Overall, these findings, albeit resulting form preclinical experiments, might build the basis for new therapeutic strategies in the upcoming years.

## Concluding Remarks

The involvement of the immune and inflammatory response in the pathogenesis of DRE and RSE are being progressively elucidated, leading to the increasing use of anti-cytokine agents in the treatment of these conditions. The experience with anti-IL-1, anti-IL-6, and anti-TNF drugs in the treatment of epilepsy is still limited and derives mostly from the observation of isolated case reports or small case series. In this work, we performed a systematic review that, despite the low evidence reached, showed promising results regarding the use of anti-cytokine agents in specific patients with DRE and RSE in terms of both efficacy and safety.

Beyond these anti-cytokine agents, there is increasing interest in the use of drugs targeting cells of adaptive immunity. The experience in the use of rituximab in RSE and DRE is fragmentary and there is a lack of a uniform regimen of treatment but this drug resulted to be effective in the major part of patients. Concerning the employment of natalizumab, the experience is limited to a single study that did not evidence a significant advantage in patients with DRE receiving the treatment.

Hopefully, preclinical research advances will allow the identification of new therapeutic strategies targeting neuroinflammation in epilepsy, and the collection of a larger number of clinical data will help in identifying those patients who will benefit from anti-cytokine treatments.

## Data Availability Statement

The original contributions presented in the study are included in the article/supplementary material, further inquiries can be directed to the corresponding author/s.

## Author Contributions

GC, GD, and AM: study design, data collection, statistical analysis, data interpretation, manuscript preparation, and literature search. TF, AO, DP, and AB: study design, data interpretation, and manuscript revision. PS, GM, SS, and RC: study design, supervision, and manuscript revision. AR: data collection, statistical analysis, data interpretation, and manuscript preparation. All authors contributed to the article and approved the submitted version.

## Conflict of Interest

The authors declare that the research was conducted in the absence of any commercial or financial relationships that could be construed as a potential conflict of interest.

## Publisher's Note

All claims expressed in this article are solely those of the authors and do not necessarily represent those of their affiliated organizations, or those of the publisher, the editors and the reviewers. Any product that may be evaluated in this article, or claim that may be made by its manufacturer, is not guaranteed or endorsed by the publisher.
